# Evaluation of the representative of using rumen fluid samples from lambs fed pelleted TMR for analysis of prokaryotic communities

**DOI:** 10.3389/fmicb.2023.1190253

**Published:** 2023-05-22

**Authors:** Zhiyuan Ma, Juwang Zhou, Ting Liu, Chen Zheng

**Affiliations:** ^1^College of Animal Science and Technology, Gansu Agricultural University, Lanzhou, China; ^2^College of Pastoral Agriculture Science and Technology, Lanzhou University, Lanzhou, China

**Keywords:** rumen fluid, rumen contents, pelleted TMR, bacteria, archaea

## Abstract

The pelleted TMR pulverized the grass during processing, which may result in more solid attached microorganisms in the filtered rumen fluid. The objective of this study was evaluating the necessity of distinguishing physical phases of rumen contents for analysis of prokaryotes communities in rumen of lambs fed pelleted TMR, considering the dissimilarity of diversity and community of bacteria and archaea between fluid and mixed rumen contents. The yield of microbial DNA, bacterial diversity, abundances of fibrolytic bacteria of phylum Fibrobacterota and Spirochaetota, as well as genus *Ruminococcus*, *Lachnospiraceae*_NK3A20, *Fibrobacter*, and F082, and abundance of archaeal *Methanimicrococcus* in rumen fluid were lower than those in mixed phase of rumen contents (*p* ≤ 0.05). In conclusion, it is necessary to consider rumen content physical phases when studying the prokaryotic community in rumen of lambs fed pelleted TMR.

## Introduction

It has been well-recognized that the microbial communities inhabiting the fluid and solid phases of rumen contents are distinguishing (Henderson et al., 2013). Bacterial communities associated with solid phase was fibrolytic phyla *Fibrobacteres* and *Firmicutes*, particularly genus *Butyrivibrio*, *Succiniclasticum* and *Lachnospiraceae*, while the predominant bacterial community member in fluid phase was *Prevotella* (Henderson et al., 2013). A hydrotrophic archaea *Methanobrevibacter* is prevalent in the rumen, accounting for 60%–95% of the total archaea (Hook et al., 2010; Ma et al., 2019). Interestingly, the structure of the archaeal community is also different between the solid and liquid phases of rumen contents, and minority archaea such as *Methanosphaera* are less abundant in the solid phase (Henderson et al., 2013; Vaidya et al., 2018).

The solid and fluid phases of rumen contents are generally separated by filtration through cheesecloth. In regular TMR for sheep, the length of the forage is generally 2–10 cm (Nielsen et al., 2017). The usual mesh size of cheesecloth used to separate rumen fluid is 250–350 μm (Ma et al., 2019), through which forage in regular TMR can be easily intercepted. However, in the pelleted TMR commonly used in lambs, the forage is pre-crushed to less than 8 mm to meet the homogenization requirements before pelleting (Malik et al., 2021). To some extent, this adds the probability of forage particles escaping into the liquid phase during squeezing filtration of cheesecloth. It is possible that the rumen fluid obtained for determination of the microbial composition could represent the rumen contents when lambs are fed pelleted TMR. Since rumen sample fractions differed substantially in terms of their physical natures and associated microorganisms, particular attention should be paid to studies involving the composition of rumen prokaryotes based on sequencing technology. Rumen solid phase is a better choice when studying rumen fibrolitic population (Gharechahi et al., 2020), while rumen fluid is undoubtedly more advantageous in animal welfare and application because of its convenience and safety (Henderson et al., 2015). The rumen contents of lambs that are fed finely ground feed are homogenized and more solid fragments escape into the liquid phase even after being filtered by gauze. Therefore, it is necessary to determine whether it is essential to distinguish between the solid and liquid phases when examining the rumen contents of lambs fed this finely ground diet.

Our hypothesis was that the characteristics of the physical form in pelleted TMR causes the solid phase of rumen contents to more easily escape cheesecloth in the process of separation. In this study, the need to distinguish physical phase of rumen contents was evaluated from the perspective of differences in prokaryotic community composition between rumen contents and rumen fluid for lambs fed pelleted TMR.

## Methods and materials

### Experimental design and animals

The animal study and sample collection were approved by the Ethics Committee of Gansu Agriculture University, Gansu, China (Approval number: GSAU-LIU-2018-02).

Under a paired design, fluid and mixed phases of rumen contents were collected from 10 *Hu* lambs (about 70-day-old, average body weight = 15.8 ± 0.76 kg) fed a same pelleted TMR. The pelleted TMR contained 5% alfalfa hay, 55.90% corn, 11% soybean meal, 1.5% whey powder, 7% expended soybean, 17% dried malt root, 1.20% limestone, 1% premix, 0.3% NaCl, and 0.1% feed attractant. All lambs had free access to water and feed.

Animals were slaughtered after a 12-h fasting period. After slaughter, about 200 g of the contents from the middle of rumen and used as the mixed phase. The liquid phase of rumen contents was achieved after filtering through four layers of cheesecloth. All samples were immersed in liquid nitrogen immediately, and then stored at −80°C until DNA extraction.

### DNA extraction

Microbial DNA were extracted from fluid and mixed phases of rumen contents using YM + SB method (Ma et al., 2020). The integrity of the DNA was validated using 1% (w/v) agarose gel electrophoresis. The DNA yields and purities were evaluated by measuring the OD_260/280_ and OD2_60/230_ ratios using a spectrophotometer (NanoDrop, Thermo Fisher Scientific, Waltham, MA, United States).

### 16S rRNA amplicon sequencing

Amplicon sequencing with single-end reads was conducted on an Ion S5 XL platform (Thermo Fisher Scientific, Waltham, MA, United States) by Novogene Co., Ltd. (Tianjing, China) according to the standard procedure of the company. In brief, microbial DNA samples were diluted to 1 ng/μL before amplifying the designated regions of 16S rRNA genes of prokaryotes. The V3-V4 bacterial 16S rRNA genes were amplified using primers of 341F (5′-CCTAYGGGRBGCASCAG-3′) and 806R (5′-GGACTACNNGGGTATCTAAT-3′) (Zakrzewski et al., 2012). The V8 regions of archaeal 16S rRNA genes were amplified using 1106F (5′-TTWAGTCAGGCAACGAGC-3′) and 1378R (5′-TGTGCAAGGAGCAGGGAC-3′) (Feng et al., 2013).

### Bioinformatic analysis

Quality control on the raw reads was performed by *vsearch* by setting -fastq_maxee *1* and -fastq_maxee_rate *0.01* (Rognes et al., 2016). Chimera sequences were detected and removed by *usearch -uchime_denovo* (Edgar, 2010). The zero-radius OTUs (ZOTUs) were identified by setting *usearch -unoise3* (Edgar, 2010). Representative ZOTU sequences were annotated with *Mothur* (Schloss et al., 2009) referring *Silva.nr. 138* for bacteria (Elmar et al., 2007) and *RIM14.6* for archaea (Seedorf et al., 2014).

The alpha diversity of ZOTUs was estimated by using observed ZOTUs and faith’s phylogenetic diversity (PD) index (Armstrong et al., 2021). The principal coordinate analysis (PCoA) was conducted based on Bray–Curtis dissimilarity matrix (Bray and Curtis, 1957) to obtain and visual principal coordinates from complex multidimensional data. The diversity calculations were performed in *R* (R Core Team, 2020), and the relevant codes is accessible at Ma (2021).

### Statistical analysis

Paired t-test were conducted using *R* with the parameter of *paired = TRUE* (R Core Team, 2020). To test for differences in overall bacterial or archaeal community between fluid and mixed phases of rumen contents, the Bray–Curtis dissimilarity matrix among sources of variation was parted, and a permutational ANOVA (PMANOVA) was performed by *vegan* with 999 permutation (Oksanen et al., 2007). All *p*-values of relative abundances of amplicon data were adjusted according to the method of Benjamini and Hochberg (1995) by *p.adjust()* function of *R* (R Core Team, 2020). A probability of *p* < 0.05 was considered to indicate a significant difference.

## Results and discussion

In our experience, rumen contents of lambs fed pelleted TMR are more viscous and more homogeneous than those of adult ruminants. This is in line with reports that pelleted TMR increases organic acid concentration (Trabia et al., 2020; Li et al., 2021) and rumen absorption capacity (Malik et al., 2021). So, we compared rumen fluid with rumen mixed contents to determine its representative.

In this study, high yield DNA was extracted from rumen fluid and rumen mixed contents. The yield of DNA extracted from adult goat rumen fluid was only one third of that in this study using the same DNA extraction method (Ma et al., 2020). This suggests that more solid contents in lambs fed pelleted TMR escaped through the cheesecloth. The microbial DNA yield in the rumen mixed content was higher than that in the rumen liquid (*p* = 0.003, Table 1). It was expected because the microbes in the solid phase are much denser than those in rumen fluid (Vaidya et al., 2018). However, neither extracted DNA from rumen fluid nor mixed contents was achieve the ideal value of 2, as indicated by OD_260/230_, the key indicators related to DNA quality, were 1.66 and 1.56, respectively. This level is close to that of DNA extracted from adult goats using the same method (Ma et al., 2020), and higher than seven out of fifteen commercially available DNA extractions methods (Henderson et al., 2013).

**Table 1:** Microbial DNA quality and alpha-diversity of fluid and mixed phase of content in rumen of lambs fed pelleted TMR starter.

Items^1^	Rumen content phase		SEM	*P*-value	
	Fluid	Mixed		Rumen content phase	Animal
DNA yield, μg/g	175	335	26.1	0.003	0.005
OD_260/280_	2.05	2.10	0.042	0.45	0.39
OD_260/230_	1.66	1.56	0.056	0.32	0.11
Bacteria					
Observed ZOTUs	1,755	1,825	18.8	0.02	0.07
PD	33.8	34.4	0.18	0.02	0.01
Archaea					
Observed ZOTUs	150	158	4.4	0.25	0.30
PD	12.3	13.5	1.23	0.48	0.57

1 Faith’s phylogenetic diversity.

A total of 1,594,679 bacterial reads and 160, 0916 archaeal reads were obtained by sequencing, with an average of 79, 733 and 80,045 reads per sample, respectively. After the denoise algorithm, the reads collapsed into 1, 911 bacterial ZOTUs and 225 archaeal ZOTUs. The bacterial alpha-diversity was higher in mixed rumen contents than in rumen fluid, as indicated by higher observed ZOTUs and PD index (Table 1, *p* = 0.02). But archaeal alpha-diversity was not influenced by physical phase of rumen contents, as indicated by similar observed ZOTUs and PD index (*p* ≥ 0.25). This result was similar to that of adult ruminants fed regular TMR (Vaidya et al., 2018), but did not align with our hypothesis. This suggests that if research needs to cover more bacteria, mixed content is a better choice.

**Figure 1:**
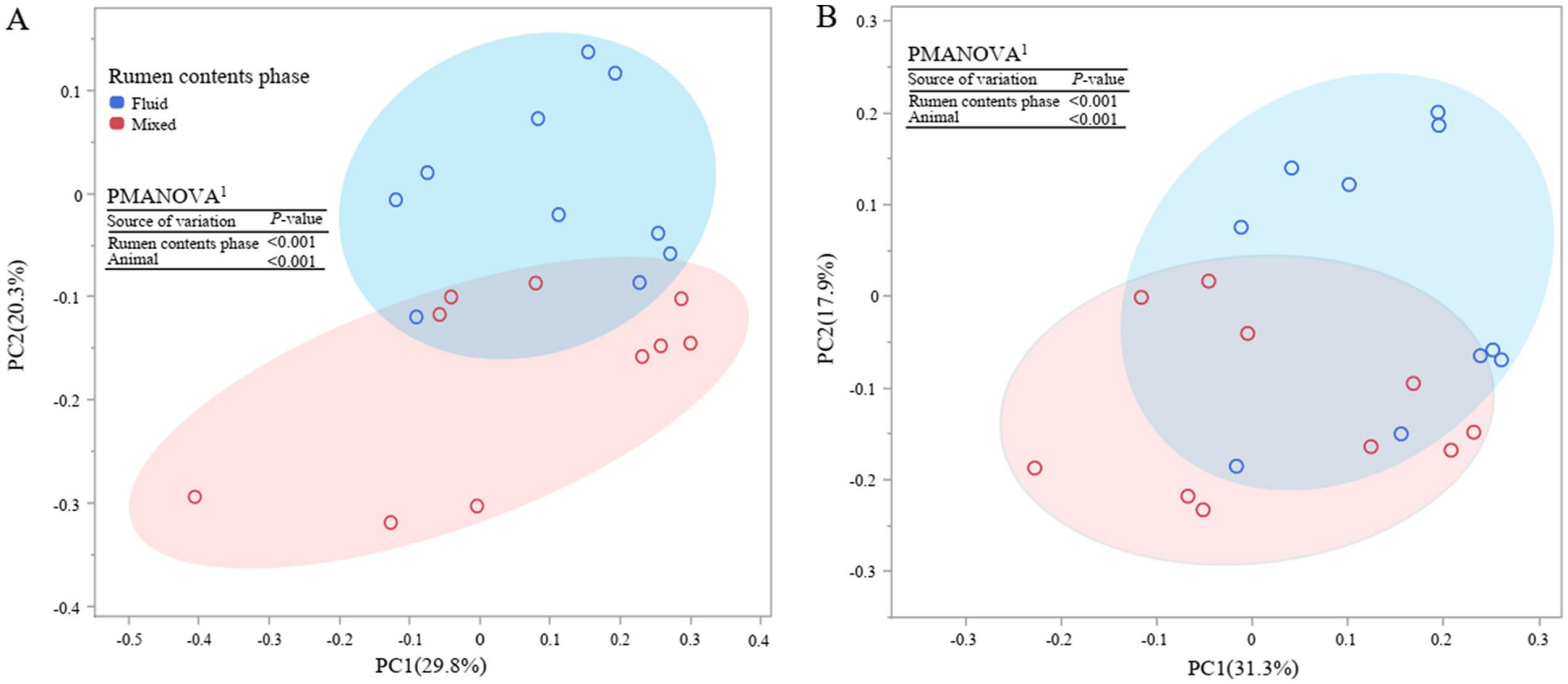
Principal coordinate analysis (PCoA) of bacterial **(A)** and archaeal **(B)** community at ZOTU level. PMANOVA, permutational multivariate analysis of variance with 999 permutations. The ellipses show contrasting prokaryotic community in mixed and liquid phases of rumen content.

**Figure 2:**
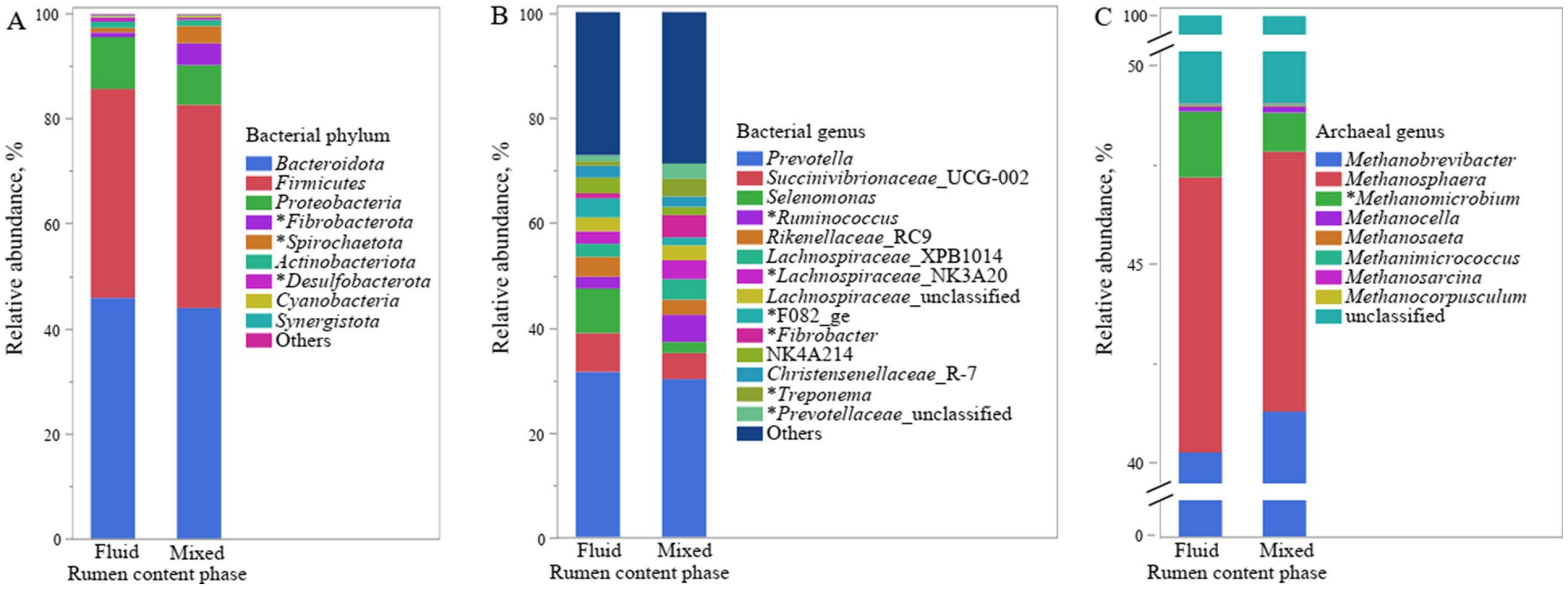
Relative abundance of bacterial phyla **(A)** and genera **(B)** and archaeal genera **(C)** in fluid and mixed phase of content in rumen of lambs fed pelleted TMR starter. The asterisk (*) before the taxonomic name indicates that its relative abundances in the mixed and liquid phases are different (*p* ≤ 0.05).

Principal coordinate analysis at ZOTU level shown both overall communities of bacteria and archaea in rumen fluid and mixed rumen contents were different (*p* < 0.001, Figure 1). Further taxonomic abundance analysis showed that the rumen fluid had lower abundances of phylum *Fibrobacterota* and *Spirochaetota*, as well as genus *Ruminococcus*, *Lachnospiraceae_*NK3A20, *Fibrobacter,* and F082, which are related to fiber degradation, than the mixed rumen contents (*p* ≤ 0.03, Figure 2; Supplementary Tables S1, S2). Populations attached to feed particles can infiltrate surface of feed plants and have more activity in degrading carbohydrate than the planktonic population (McAllister et al., 1994). It is well recognized that the population of fibrolytic bacteria on rumen fluid is much lower than on forage grass (De Mulder et al., 2017; Vaidya et al., 2018). Unfortunately, the relative abundances of fibrolytic bacteria in rumen fluid was not representative of the rumen contents, although pelleted TMR greatly reduced the size of the forage.

We detected that 99.9% of the archaea were *Euryarchaeota*, so they were not analyzed at the phyla level. Archaea in the rumen are hydrogen-trophic methanogens that usually coexist with hydrogen-producing microorganisms such as fibrolytic bacteria (Czerkawski et al., 1972). The second largest archaeal genus *Methanosphaera* in the rumen has been reported to be more abundant in rumen fluid than in solid rumen contents (Henderson et al., 2013; Vaidya et al., 2018). However, we only observed a numerical change (*p* = 0.87; Figure 2 and Supplementary Table S3). Our comparison of rumen fluid with mixed rumen contents rather than solid phase may have reduced this discrepancy to some extent. We observed that archaeal genus *Methanimicrococcus* was less abundant in rumen fluid than in mixed rumen contents (*p* = 0.01). Genus *Methanimicrococcus* has been reported to decrease in abundance with the increase of dietary forage (Huo et al., 2020), suggesting that it may be symbiotic with planktonic hydrogen-producer. Such speculation was contradicted with the lower relative abundance of genus *Methanimicrococcus* in rumen fluid than in mixed rumen contents. More research is needed to understand this phenomenon.

In conclusion, the yield of microbial DNA, bacterial diversity, abundance of fibrolytic bacteria, and abundance of archaeal *Methanimicrococcus* in rumen fluid were lower than those in mixed rumen contents. Therefore, this study emphasizes the need for careful consideration of sample collection methods in rumen microbial studies, especially in young ruminants like lambs.

## Data availability statement

The datasets presented in this study can be found in online repositories. The names of the repository/repositories and accession number(s) can be found in the article/Supplementary material.

## Ethics statement

The animal study was reviewed and approved by Ethics Committee of Gansu Agriculture University.

## Author contributions

ZM: conceptualization, investigation, software, data curation, writing, visualization, and funding acquisition. JZ: investigation, methodology, and data curation. TL: conceptualization, data curation, supervision, funding acquisition, and project administration. CZ: investigation, writing—review and editing, and supervision. All authors contributed to the article and approved the submitted version.

## Funding

The present study was financially supported by National Natural Science Foundation of China (31860656), China Postdoctoral Science Foundation (2022M711452), the Youth Science and Technology Fund Program of Gansu province (20JR10RA553) and Discipline Team Project of Gansu Agricultural University (GAU-XKTD-2022-20).

## Conflict of interest

The authors declare that the research was conducted in the absence of any commercial or financial relationships that could be construed as a potential conflict of interest.

## Publisher’s note

All claims expressed in this article are solely those of the authors and do not necessarily represent those of their affiliated organizations, or those of the publisher, the editors and the reviewers. Any product that may be evaluated in this article, or claim that may be made by its manufacturer, is not guaranteed or endorsed by the publisher.
