# Supplementary material for: Evaluation of the representative of using rumen fluid samples from lambs fed pelleted TMR for analysis of prokaryotic communities
Source: Front Microbiol. 2023 May 22;14:1190253. doi: 10.3389/fmicb.2023.1190253 (PMC10240055; doi:10.3389/fmicb.2023.1190253)
Supplement: Supplementary file 1 [file Table_1.DOCX]

Table S1 Relative abundance (%) of bacterial phyla in fluid and mixed phase of content in rumen of lambs fed pelleted TMR starter

| Item | Rumen content phase | | *P*-value | | |
| --- | --- | --- | --- | --- | --- |
|  | Fluid | Mixed | SEM | Rumen content phase | Animal |
| *Bacteroidota* | 45.9 | 44.0 | 5.61 | 0.79 | 0.09 |
| *Firmicutes* | 39.8 | 38.6 | 7.30 | 0.92 | 0.04 |
| *Proteobacteria* | 9.85 | 7.56 | 3.455 | 0.60 | 0.02 |
| *Fibrobacterota* | 0.89 | 4.14 | 0.744 | <0.001 | 0.22 |
| *Spirochaetota* | 0.88 | 3.28 | 0.372 | <0.001 | 0.09 |
| *Actinobacteriota* | 1.17 | 1.16 | 0.278 | 0.98 | 0.02 |
| *Desulfobacterota* | 0.81 | 0.38 | 0.204 | 0.04 | 0.09 |
| *Cyanobacteria* | 0.26 | 0.40 | 0.123 | 0.31 | 0.18 |
| *Synergistota* | 0.19 | 0.18 | 0.024 | 0.72 | 0.002 |
| Others | 0.17 | 0.14 | 0.030 | 0.28 | 0.14 |

Table S2 Relative abundance (%) of bacterial genera in fluid and mixed phase of content in rumen of lambs fed pelleted TMR starter

| Item | Rumen content phase | | *P*-value | | |
| --- | --- | --- | --- | --- | --- |
|  | Fluid | Mixed | SEM | Rumen content phase | Animal |
| *Prevotella* | 31.4 | 30.1 | 4.99 | 0.70 | 0.11 |
| *Succinivibrionaceae*_UCG-002 | 7.38 | 4.97 | 3.143 | 0.40 | 0.06 |
| *Selenomonas* | 8.48 | 2.11 | 5.74 | 0.21 | 0.22 |
| *Ruminococcus* | 2.23 | 5.16 | 0.602 | <0.001 | 0.07 |
| *Rikenellaceae*_RC9 | 3.78 | 2.88 | 0.684 | 0.17 | 0.07 |
| *Lachnospiraceae*_XPB1014 | 2.50 | 3.89 | 1.111 | 0.17 | 0.06 |
| *Lachnospiraceae*_NK3A20 | 2.37 | 3.58 | 0.518 | 0.02 | 0.06 |
| *Lachnospiraceae*_unclassified | 2.63 | 2.87 | 0.724 | 0.70 | 0.03 |
| F082_ge | 3.66 | 1.49 | 1.017 | 0.03 | 0.14 |
| *Fibrobacter* | 0.92 | 4.25 | 0.768 | <0.001 | 0.22 |
| NK4A214 | 3.00 | 1.54 | 0.899 | 0.09 | 0.21 |
| *Christensenellaceae*_R-7 | 2.20 | 1.99 | 0.506 | 0.64 | 0.06 |
| *Treponema* | 0.89 | 3.34 | 0.381 | <0.001 | 0.09 |
| *Prevotellaceae*_unclassified | 1.17 | 2.92 | 0.417 | <0.001 | 0.06 |
| Others | 27.2 | 28.8 | 2.65 | 0.51 | 0.11 |

Table S3 Relative abundance (%) of archaeal genera in fluid and mixed phase of content in rumen of lambs fed pelleted TMR starter

| Item | Rumen content phase | | *P*-value | | |
| --- | --- | --- | --- | --- | --- |
|  | Fluid | Mixed | SEM | Rumen content phase | Animal |
| *Methanobrevibacter* | 40.2 | 41.2 | 3.25 | 0.83 | 0.28 |
| *Methanosphaera* | 6.92 | 6.56 | 1.550 | 0.87 | 0.32 |
| *Methanomicrobium* | 1.65 | 0.98 | 0.449 | 0.31 | 0.16 |
| *Methanocella* | 0.12 | 0.15 | 0.013 | 0.14 | <0.001 |
| *Methanosaeta* | 0.03 | 0.03 | 0.001 | 0.86 | <0.001 |
| *Methanimicrococcus* | 0.02 | 0.03 | 0.003 | 0.01 | <0.001 |
| *Methanosarcina* | 0.01 | 0.01 | 0.0008 | 0.27 | <0.001 |
| *Methanocorpusculum* | 0.002 | 0.0007 | 0.00077 | 0.09 | 0.25 |
| Unclassified | 50.9 | 50.9 | 2.82 | 0.99 | 0.18 |
